# Sociodemographic Factors Attributed to the Double Burden of Malnutrition in Urban Bangladesh

**DOI:** 10.3390/nu18010135

**Published:** 2025-12-31

**Authors:** Md. Saimul Islam, Nick Townsend, Afrin Iqbal, Nabila Mahmood, Abdullah Mamun, Aliya Naheed

**Affiliations:** 1 Non-Communicable Diseases, Nutrition Research Division, icddr,b, Dhaka 1000, Bangladesh; saimul.islam@icddrb.org (M.S.I.); nabila.mahmood@icddrb.org (N.M.); 2 School for Policy Studies, University of Bristol, Clifton BS8 1TZ, UK; nick.townsend@bristol.ac.uk; 3 Maternal and Child Health Division, icddr,b, Dhaka 1000, Bangladesh; afrin.iqbal@icddrb.org; 4 Faculty of Humanities, Arts and Social Sciences, The University of Queensland, Brisbane, QLD 4072, Australia; a.mamun@uq.edu.au

**Keywords:** malnutrition, children, adolescents, mother-child pairs and Bangladesh

## Abstract

**Background:** There is a high prevalence of the double burden of malnutrition (DBM) in children and adolescents in South Asia. This research aims to explore which sociodemographic factors are attributed to DBM in urban Bangladesh, a South Asian country. **Methods:** We conducted secondary analyses of data obtained from the national survey of childhood obesity among school-age children in Bangladesh (2012–2013). The sample includes 4140 children (aged 5–9 years) and adolescents (10–19 years) randomly recruited from the city corporation (urban) areas in all administrative divisions. At the population level, DBM was defined as the coexistence of underweight and overweight/obesity among children and adolescents. At the household level, DBM was defined as maternal underweight co-occurring with child overweight/obesity within the same mother-child dyad. A multivariable logistic regression model was fitted to estimate odds ratios and 95% confidence intervals. A rapid policy review was conducted to understand the implication of the results obtained from the analysis. **Results:** The prevalence of DBM at the population level was 45.2% (95% CI: 42.5–45.5%), ranging between 40.0% and 47.6% across seven divisions (*p* < 0.001). At the household level, DBM prevalence was 16.6% (95% CI: 14.7–18.7%), ranging between 14.0% and 19.0% across seven divisions (*p* = 0.015). At the population level, DBM odds were 56% higher among younger children (5–9 years) than adolescents (10–19 years) (OR: 1.56; 95% CI: 1.37–1.78), and this association was found in four divisions. At the household level (mother-child pairs), DBM odds were 64% higher in younger children than adolescents (OR: 1.64; 95% CI:1.38–1.95); and higher in children living at a lower-middle socioeconomic status (SES) and middle SES, than upper SES. The policy review revealed that Bangladesh has made substantial commitments to improve nutrition; however, reference to DBM is absent from policy documents. **Conclusions:** The prevalence of DBM is high among children in urban areas in Bangladesh, disproportionately affecting younger children and households with low SES. In the current policy space, Bangladesh should revise national nutrition frameworks to recognize DBM as a public health priority and implement region-sensitive strategies for preventing and reducing malnutrition among school-aged children.

## 1. Introduction

The double burden of malnutrition (DBM), defined as the coexistence of undernutrition and overnutrition, represents a major public health challenge among children and adolescents, i.e., individuals aged 5 to 19 years, globally [1,2]. As of 2022, an estimated 390 million children and adolescents worldwide were overweight, while around 190 million suffered from undernutrition [3,4]. DBM can occur at the population level, within households (for example, maternal underweight coexisting with child overweight/obesity), or even within a single individual [5,6,7,8]. Different forms of the DBM disproportionately affect about one-third of populations in low- and middle-income countries (LMICs) [6].

The 2020 Lancet Commission on DBM reported that South Asia mirrors these global trends, as the region continues to experience a persistent burden of undernutrition alongside a rapid increase in childhood overweight and obesity [6]. In particular, urban areas experience a higher prevalence of DBM than rural settings [9,10]. Rapid urbanization has shifted dietary patterns toward increased consumption of processed food and a more sedentary lifestyle, which increases the risk of overnutrition among children. At the same time, low household income limits access to adequate and diverse diets, leading to undernutrition [11,12]. Consequently, the coexistence of under and overnutrition has become increasingly prevalent across the region.

Within this regional context, Bangladesh is undergoing substantial socio-demographic transitions, including a surge in urban development and changes in nutritional status [13,14]. Low parental education and socio-economic inequalities also influence the nutritional landscape among children in urban settings [15]. Evidence suggests that undernutrition remains prevalent among poorer households, whereas overweight and obesity are increasingly observed in wealthier families [16,17]. National surveys report that 15.8% of urban households experience DBM, typically characterized by mothers with overweight paired with children under 5 years old who are stunted [18]. In urban Dhaka, one study reported that 16% of children and adolescents were underweight, while 28% were overweight [19].

Despite this emerging trend, data on DBM are limited to school-aged children and women of reproductive age in urban settings [19,20]. Although the prevalence of DBM has been reported to vary across different socioeconomic statuses (SES) in Bangladesh, there is a lack of DBM prevalence estimates at the population or household levels [21,22]. Furthermore, factors attributed to the high burden of DBM are also not well understood. Such evidence would be crucial for identifying the most vulnerable groups, those with a high burden of malnutrition, at national and regional levels [18,19,20,21].

The National Nutrition Services (NNS) in Bangladesh coordinates nutrition policy and integrates services into primary health care through training, capacity-building, supervision, and routine monitoring under the Health, Population and Nutrition Sector Program [23]. Core nutrition services include growth monitoring, feeding counseling and micronutrient supplementation for children, nutrition counseling and breastfeeding support, and iron–folic acid supplementation for women, which are delivered through community health workers [24]. In Bangladesh, mothers are typically the principal caregivers for child growth and wellbeing, making mother–child dyads a relevant unit for understanding DBM. However, it remains unclear whether current nutrition policies recognize the burden of DBM or provide guidance for addressing the emerging challenge.

To address the evidence gap, national survey data on urban children and adolescents (5–19 year) in Bangladesh were used for secondary analysis. This study aims to identify socio-demographic factors associated with DBM and assess whether existing nutrition policies adequately address this challenge.

## 2. Materials and Methods

### 2.1. Study Design and Participants

**Figure 1:**
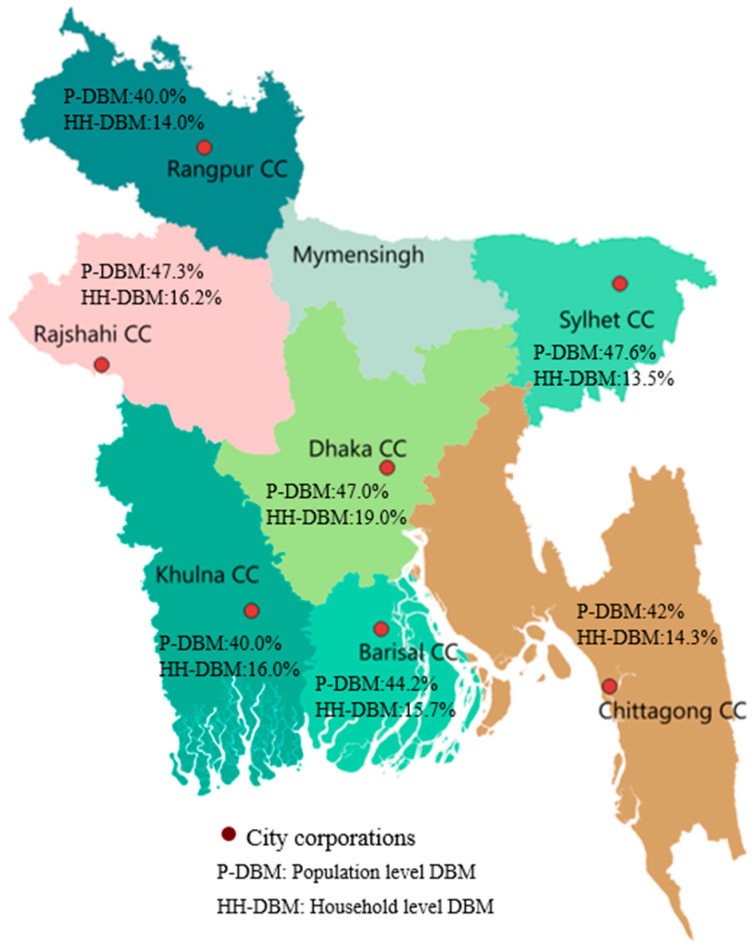
Locations of city corporations in Bangladesh and division-level DBM prevalence (population and household). *(Note: Mymensingh division was included under Dhaka division before 2013)*.

We analyzed data from the nationwide survey on childhood obesity, which was conducted between January and June 2013 by the International Centre for Diarrhoeal Disease Research, Bangladesh (PR#13024) sponsored by the NNS. The survey was conducted in seven city corporation (CC) areas, including Dhaka, Chattogram, Rajshahi, Sylhet, Khulna, Barisal and Rangpur divisions (Table 1 and Figure 1). Dhaka represents the oldest city corporation, established in 1864, and Rangpur is the newest city corporation, established in 2012 [25]. Thirty wards (the lowest administrative unit in a CC) out of the total were randomly selected from each CC, and all households with a child aged 5–19 years and their biological mothers were listed in order to develop the sampling frame. From each ward, twenty children were randomly selected using the improved WHO Expanded Programme on Immunization (EPI) cluster-sampling approach [26]. Written consent was obtained from parents or guardians of all participants, and assent obtained from children aged 10–19 years. Any child who lived in a study area for less than six months or did not have a biological mother or had a mother who was too sick to provide consent was excluded from the survey.

Trained research staff used a structured questionnaire to collect data from children on age, sex, school grade completed, and parents’ information, including education and socio economic status (ownership of durable assets, materials used for the roof, walls, and floor, access to electricity, type of toilet facilities, drinking water sources and types of cooking fuels). In the national survey, the height of children and mothers were measured using a wooden stadiometer, and weight was measured using a Tanita HD-351 digital scale (Tanita Corporation, Tokyo, Japan) [27]. Data quality was checked with 5% of the respondents within 24 h of data collection. All data forms were transported to the designated data management team in icddr,b every week for data entry.

The BMI of children was estimated following the International Obesity Task Force (IOTF) reference curves using the standard formula: weight (kg)/height (m^2^), applying the age and sex specific BMI cut-off values to classify children and adolescents (aged 5–18 years) into underweight (<5th percentile), normal weight (5th–84th percentile), overweight (85th–94th percentile), and obese (≥95th percentile) [28,29,30]. The BMI of mothers was classified into underweight (<18.5 kg/m^2^), normal weight (≥18.5 and <23.0 kg/m^2^), overweight (≥23.0 and <27.5 kg/m^2^), and obese (≥27.5 kg/m^2^) using the WHO recommended Asian BMI cut-off values [31].

### 2.2. Data Acquisition and Data Management for Secondary Analyses

The full database was obtained from icddr,b and the data were managed by a qualified group of data managers. Age of children was categorized in to two groups: (i) Those 5 to 9 years of age were defined as younger children and (ii) 10 to 19 years of age defined as adolescents, following WHO guidelines [32]. Educational status was categorized into four groups: (1) never attended a school, (2) below primary (completed 1 to 4 grades or attended a pre-school), (3) completed primary (completed 5 to 9 grades), (4) completed secondary (completed 10th to 12th grade) and (5) completed higher secondary (completed 13th grade or above). Low education was defined for those who never attended a school or had an education below primary. The father’s occupation was categorized as service, business, or manual workers (rickshaw/van, puller/transport, worker/migrant, worker/agriculture). The mother’s occupation was categorized as housewife or employed (service, business, and manual labor). Wealth index (WI) was calculated using principal component analysis (PCA) following the standard guidelines of the Demographic and Health Survey [33]. The WI variable was then divided into five quintiles to define socioeconomic status: lower quintile (1st 20% of WI), lower-middle quintile (2nd 20% of WI), middle quintile (3rd 20% of WI), upper-middle quintile (4th 20% of WI), and upper quintile (5th 20% of WI).

### 2.3. Data Analysis

At the population level, DBM was categorised as the co-occurrence of undernutrition and overweight/obesity among children and adolescents within the same population [34]. To ensure clarity, we first estimated the prevalence of undernutrition and the prevalence of overweight/obesity as separate measures. Population-level DBM was then defined as present when both forms of malnutrition occurred within the study population. At the household level, DBM was categorised as the coexistence of maternal overweight/obesity and child undernutrition within the same household [35,36]. For analytical purposes, we created separate binary variables for population level and household level DBM, coded as 1 if DBM was present and 0 otherwise. These variables were subsequently used for descriptive and multivariable analysis.

Descriptive statistics were used to summarize the sociodemographic characteristics of the study participants and presented as frequencies and percentages (categorical variables), or means with standard deviations (SD) (continuous variables). The prevalence of DBM at both the population and household levels were reported by percentage with 95% confidence intervals (CIs). Comparisons between DBM and key socio-demographic factors, including age of children, maternal education, and socioeconomic status (SES) were assessed using chi-square (χ^2^) tests. A Spearman’s rank correlation test was performed to assess the relationship between DBM and socioeconomic status.

Covariates included in the analyses were selected based on prior evidence of association with DBM [19,37] and exploratory assessment of potential predictors. Multivariable logistic regression models were applied after adjusting for the child’s age, sex, father’s occupation, and SES. Adjusted odds ratios (AORs) with 95% CIs were reported for modeling DBM with socio-demographic characteristics and socio-economic conditions. Region-specific analyses were performed to explore variations of the prevalence of DBM and its socio-demographic characteristics across the administrative divisions. All statistical tests were two-tailed. In bivariate analysis, a *p*-value < 0.1 was considered statistically significant to identify candidate variables for inclusion in the multivariable model. For assessing the association between DBM and participant characteristics in the multivariable regression model, a *p*-value < 0.05 was considered significant to determine independent association. Model fit was assessed using Hosmer-Lemeshow goodness-of-fit tests. A selection of variables were confirmed in the multivariable model after variance inflation factors were calculated to check for multicollinearity among predictors. All analyses incorporated the complex survey design, including clustering at the ward level, stratification by City Corporation (CC), and post-stratification sampling weights. Sampling weights were constructed to account for unequal probabilities of selection across the seven CCs, and were normalized to have a mean of 1 before use in the analyses. Weighted estimates therefore represent the population of children and adolescents aged 5 to 19 years living in urban Bangladesh (Supplementary File). Average marginal effects (AME) with 95% confidence intervals (CIs) were computed for the child age, mother’s education, father’s education, and socioeconomic status (SES). All models were adjusted for relevant covariates. Standard errors were clustered at the appropriate survey level. AMEs were derived to provide interpretable effect estimates representing the change in predicted probability of DBM associated with a one-unit change in each predictor. Analyses were conducted using standard post-estimation marginal effects procedures. All analyses were based on complete-case data; no missing values were observed for the variables included in the analyses. IBM SPSS Statistics for Windows, Version 20.0 (IBM Corp., Armonk, NY, USA), version 20.0 and R programming language were used for all the statistical analyses.

### 2.4. Rapid Policy Review

A rapid policy review was conducted to explore the extent to which existing policy documents addressed DBM during the major policy changes introduced through the 6th Health, Population, and Nutrition sector programme of Bangladesh (2011–2016), which prioritized nutrition. As the survey was also conducted during this timeframe, the review focused on policy documents published between 2010 and 2016. We systematically searched ministerial websites including the Ministry of Health and Family Welfare, Ministry of Women and Children Affairs, Ministry of Food, and other relevant ministries to identify policies related to nutrition and health. The search used a predefined list of keywords and Boolean operators, including “nutrition, “double burden”, “child health,” “adolescent health,” “maternal nutrition” and “obesity” applied both individually and in combination.

Documents were eligible for inclusion if their titles or abstracts indicated relevance to the health or nutrition of children, adolescents, or women and if they were officially issued within the target period. Documents were excluded if they were draft versions without endorsement, operational guidelines lacking policy relevance, or materials unrelated to nutrition or health. Eligible documents were then screened for explicit or implicit references to DBM and for policy measures that could address both undernutrition and overnutrition. Key information from included documents was extracted systematically using a structured template. A thematic analysis approach was applied to identify policy objectives, strategic actions, and intersectoral linkages related to DBM and highlight gaps or inconsistencies across sectors.

### 2.5. Ethics

The national survey of childhood obesity was originally approved by the Ethical Review Committee of icddr,b. Written consent was obtained from the mothers of children 5 to 17 years and children 18 years or older. Assent was obtained from children 10–17 years. This study conducted analyses on already collected data from the national survey; hence, no additional ethical approval was required. The data used for this secondary analysis were fully de-identified prior to access. All files were securely stored on password protected servers with restricted access to ensure participant confidentiality and data integrity.

### 2.6. Role of the Funding Source

The funder of the study had no role in study design, data collection, data analysis, data interpretation, or writing of the report.

## 3. Results

### 3.1. Characteristics of the Participants

The national survey included a total of 4140 children including ~600 children and their mothers from each CC in a division. The mean age of children was 11 years (SD: 4), including 50.3% girls and 66.8% adolescents (10–19 years). Nearly half of the children had an education below primary (47.0%), 34.9% had completed primary, 11.5% completed secondary and 6.6% never attended a school. Among mothers of the children, 13.3% had never attended a school, 10.4% had an education below primary (23.7%), 39.7% completed primary, and 17.4% completed secondary education. Among fathers of the children, 14.1% had never attended a school, 23.6% had an education below primary, 32.2% completed primary education, and 44.1% had completed secondary education. Occupation of the fathers were business (43.4%), service (33.4%), and manual labor (21.5%), while 91% of the mothers of the children were housewives (Table 2).

### 3.2. Prevalence of Population Level DBM and Variations Across Divisions

Among children and adolescents, 27.2% were underweight and 18.0% were overweight/obese. The population level DBM, reflecting the coexistence of both forms of malnutrition, was 45.2% (95% CI: 42.6–47.8%). The prevalence of DBM was significantly higher amongst children than adolescents (51.3% vs. 40.3%; *p* < 0.001). A higher DBM prevalence was found amongst children of mothers with a low education compared to children of mothers who had completed secondary education (48.3% vs. 43.1%; *p* = 0.007). Similarly, children whose fathers had a low education had a higher prevalence of DBM than children whose fathers had completed secondary education (47.7% vs. 41.8%; *p* = 0.011). The highest prevalence of DBM was observed in the Sylhet division (47.5%) followed by Rajshahi (47.3%), Dhaka (47.0%), Barisal (44.2%), Chattogram (42.0%), Khulna (40.0%), and Rangpur (40.0%). No significant variations in DBM were observed by sex and parents’ occupation (Table 3 and Figure 1).

### 3.3. Factors Attributed to Population Level DBM and Variations Across Divisions

**Figure 2:**
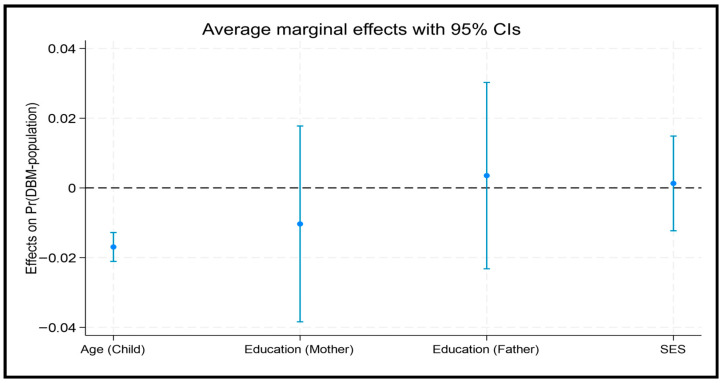
Average marginal effect of socio-demographic characteristics on DBM at population level.

Table 4 presents the adjusted odds ratios (ORs) of the relationship between DBM among children and adolescents (5–19 years) at the population level across Bangladesh. Overall, younger children had significantly higher odds of DBM than adolescents (OR = 1.56; 95% CI: 1.37–1.78). The AME for age (AME = −0.0169, *p* < 0.001) indicates that for each additional year of age, the predicted probability of DBM decreases by approximately 1.7% (Figure 2). At the regional level, the relationship between age and DBM was significant in Barisal (OR = 1.83; 95% CI: 1.29–2.60), Khulna (OR = 1.57; 95% CI: 1.09–2.27), Rajshahi (OR = 2.28; 95% CI: 1.60–3.25), and Rangpur (OR = 1.77; 95% CI: 1.22–2.56), which was not observed in other divisions (Table 4 and Figure 1).

Overall, no relationship was observed between DBM and SES. However, in Sylhet division, the odds of DBM were three times higher in lower SES (OR = 3.21; 95% CI: 1.16–8.90), two times higher in lower-middle SES (OR = 2.06; 95% CI: 1.12–3.80), and 74% higher in middle SES (OR = 1.74; 95% CI: 1.07–2.82) compared to upper SES. In Dhaka, by contrast, children from upper-middle SES had lower odds of DBM than upper SES (OR = 0.59; 95% CI: 0.37–0.93) (Table 4)**.**

While no relationship was observed between DBM and parental education overall, the regional analysis demonstrated that children in Khulna whose fathers had low education (OR = 0.48; 95% CI: 0.26–0.86) or completed primary (OR = 0.59; 95% CI: 0.36–0.97) had lower odds of DBM compared with those children whose fathers completed secondary education. Conversely, children whose fathers had low education had nearly two times higher odds of DBM (OR = 1.96; 95% CI: 1.11–3.48) compared to children whose fathers completed secondary level in Rangpur division. No significant relationship was observed in the other divisions (Table 4).

Maternal education demonstrated a protective relationship in Sylhet, where children of mothers who completed primary education had lower odds of DBM than those children whose mothers completed secondary education (OR = 0.60; 95% CI: 0.38–0.97), which was not observed in other divisions (Table 4). The average marginal effects of socio-demographic characteristics on DBM were consistent across the divisions, exhibiting similar variations at the population level. (Supplementary File, Figure S1)

### 3.4. Prevalence of DBM Among Mother-Child Pairs at the Household Level

The prevalence of household level DBM was 16.6% (95% CI: 14.7–18.7%), but higher amongst younger children than adolescents (20.0% vs. 13.4%; *p* = 0.04). Prevalence varied substantially by region, ranging from 28.1% in Sylhet to 14.7% in Rangpur (*p* < 0.001). DBM was prevalent in households where mothers (28.8% vs. 18.8%; *p* = 0.032) or fathers (26.9% vs. 19.4%; *p* = 0.003) had low educational attainment compared with households in which parents had completed secondary education. DBM prevalence was also higher in households where fathers were employed in service (26.3%) or business (25.1%) compared to households in which fathers were engaged in manual labor (16.0%; *p* < 0.001). DBM was also more prevalent in the upper SES than the lower-middle SES households (18.0% vs. 12.0%; *p* < 0.001) (Table 5).

### 3.5. Factors Attributed to Household Level DBM and Regional Variations

**Figure 3:**
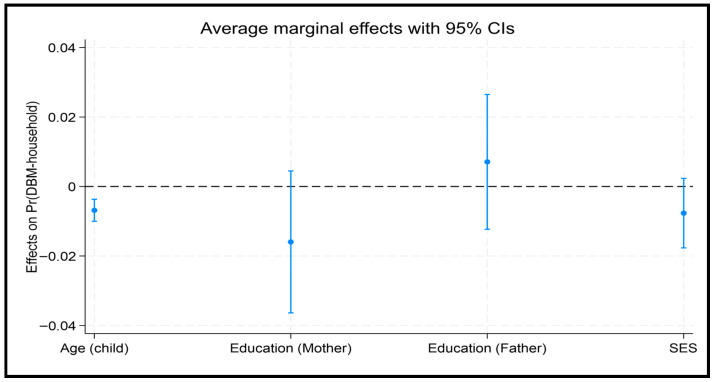
Average marginal effect of socio-demographic characteristics on DBM at household level (Mother-Child Pair).

At the national level, households with a younger child had 64% higher odds of DBM than those with a child aged 10–19 years (OR = 1.64; 95% CI: 1.38–1.95). The AME for age (AME = −0.0068478, *p* < 0.001) indicates that for each additional year of age, the predicted probability of DBM decreases by approximately 0.69% (Figure 3). Across divisions, younger children remained at significantly higher odds in Dhaka (OR = 1.83; 95% CI: 1.17–2.86), Barisal (OR = 1.99; 95% CI: 1.22–3.33), and Rajshahi (OR = 1.96; 95% CI: 1.25–3.06), whereas no significant association was observed elsewhere (Table 5 and Figure 1).

The odds of DBM were higher for children in lower-middle SES (OR = 1.41; 95% CI: 1.03–1.93) and middle SES households (OR = 1.49; 95% CI: 1.12–2.03) than upper SES households. SES-related disparities were observed in Sylhet and Chattogram. In Sylhet, the odds of DBM were more than twice as high among children who belonged to lower-middle (OR = 2.64; 95% CI: 1.13–6.18) and middle SES (OR = 2.62; 95% CI: 1.32–5.19) households compared to children from upper SES. In Chattogram, children from lower SES (OR = 2.75; 95% CI: 1.05–7.20) and lower-middle SES (OR = 2.51; 95% CI: 1.16–5.47) households had significantly higher odds of DBM than those from upper SES households. However, no relationship was observed in other divisions (Table 5).

In adjusted models, parental education was not significantly associated with DBM. In Khulna, children whose fathers had low education had significantly lower odds of DBM compared with children whose fathers completed secondary education (OR = 0.38; 95% CI: 0.17–0.82), whereas having a mother with low education was correlated with four times higher odds of DBM (OR = 4.26; 95% CI: 1.81–10.0) compared with children whose mother had completed secondary education. No significant associations with parental education were observed in the other divisions (Table 6). In summary, socio-demographic factors showed similar average marginal effects on household-level DBM across all divisions (Supplementary File, Figure S2).

### 3.6. Rapid Policy Review Findings

A review of seven national policy and action documents revealed that undernutrition remains the dominant focus, particularly among children, adolescent girls, and pregnant or reproductive-age women. Early policies such as the National Health Policy, 2011 addressed undernutrition almost exclusively, with no consideration of overweight, obesity, or the DBM.

Since 2015, more recent policies, including the National Nutrition Policy, 2015 and the Bangladesh National Food and Nutrition Security Policy (2021–2030), acknowledge the emergence of overweight, obesity, and nutrition related non-communicable diseases. However, these acknowledgements are largely descriptive; none of the policies explicitly reference DBM or double-duty actions, and interventions for overnutrition are largely implemented in parallel to undernutrition programmes rather than integrated with them.

Several interventions embedded within these policies have potential double-duty effects, including exclusive breastfeeding and complementary feeding, micronutrient supplementation (iron and folic acid) for mothers and children, school feeding programme, nutrition education, and regulation of processed food marketing. While these interventions indirectly target both under- and overnutrition, the lack of explicit DBM framing constrains coherent cross-sectoral implementation and systematic monitoring.

Policy-specific observations reveal that maternal and adolescent health strategies (2017–2030) provide the greatest potential to address household-level DBM, particularly through mother-child nutrition interventions and school-based programmes. School-focused policies (2019) combine nutrition provision with education and retention objectives, but risk neglecting obesity prevention if calorie-centric interventions are not balanced with quality nutrition. Recent regulatory measures and nutrition education targeting adolescents (2021–2030) could address both forms of malnutrition at the population level but require multi-sectoral coordination to maximize impact.

Across policies, key operational gaps include (1) the lack of explicit DBM or double-duty action frameworks, limiting integrated planning; (2) predominance of single-form interventions, creating programmatic silos; (3) weak monitoring and evaluation systems for dual outcomes; and (4) limited household-level interventions addressing overlapping maternal-child malnutrition.

Implications: Policies show a gradual shift from single-burden to multi-burden awareness, but current frameworks stop short of operationalizing integrated strategies. Formal incorporation of DBM objectives, strengthened cross-sectoral coordination, and monitoring for both under- and overnutrition are essential to address the emerging nutritional landscape in Bangladesh. The results from the rapid review have been presented in Table 7.

## 4. Discussion

This study provides the first national-level estimates of the DBM and its regional variations in urban Bangladesh at both population and household levels. The findings reveal a complex interplay of socio-demographic characteristics and maternal factors contributing to DBM. Nearly half of the children and adolescents experienced DBM at the population level, with younger children more affected than adolescents. At the household level, one-fourth of the mother-child pairs were affected, with a higher burden observed among mothers with a higher education and those living in a higher SES group. Notably, mothers with a low education in middle to upper SES households exhibited a higher burden of malnutrition among younger children compared to adolescents.

This high prevalence highlights the substantial burden of both undernutrition and overnutrition among children and adolescents, consistent with findings from other low- and middle-income countries [38,39]. The DBM estimate in Bangladesh is higher than those reported in Ethiopia [40], and is rapidly emerging in South and Southeast Asia [41]. This dyadic approach provides a culturally and contextually appropriate understanding on how maternal nutrition and caregiving practices influence child nutritional outcomes in Bangladesh. These findings underscore the need to identify vulnerable populations experiencing the highest burden across socio-demographic characteristics and regional factors.

Younger children showed a substantially higher likelihood of experiencing DBM than adolescents, a pattern evident across all regions in Bangladesh. Early childhood is a critical stage of growth and development, making them more vulnerable to the adverse effects of malnutrition [42]. These younger children are particularly susceptible to micronutrient deficiencies, which can compromise immune function and increase the likelihood of malnutrition [43]. The findings may be explained by the fact that rapid shifts in dietary practices and lifestyle patterns often occurring unevenly within households can create mismatched nutritional exposures between mothers and young children. Given these complexities, it is essential to explore the role of parental education in improving the nutritional status of children and mitigating the risks associated with DBM.

Our findings indicate that the relationship between parental education and DBM reflects deeper socioeconomic and structural dynamics than simple differences in prevalence. Studies from Bangladesh and other LMICs show that higher maternal education is associated with a reduction in child undernutrition, yet in some contexts, it simultaneously predicts higher risk of childhood overnutrition [44,45,46]. Educated mothers in high-income households may have greater exposure to unhealthy diets and processed foods [47]. These seemingly contradictory patterns can be understood through the nutrition transition theory and the social determinants of health. Therefore, maternal education along with the socioeconomic position of the household may contribute to the increasing burden of DBM at both the population and household levels.

Our research further suggests that children whose fathers were engaged in business and service occupations were more likely to have a higher burden of DBM compared to those children whose fathers were manual workers. Similar trends have been reported in other LMICs settings, where households with relatively better income sources often experienced a dual burden of malnutrition, due to a nutritional transition [48]. Increased income may lead to greater consumption of energy-dense, processed foods contributing to overweight and obesity, while underlying child undernutrition persists due to poor dietary diversity or inadequate feeding practices [6]. In contrast, families dependent on manual labor may have limited resources, but their diets are less likely to include calorie-rich processed foods, potentially reducing the risk of overweight [49]. This underscores the complexity of socio-economic drivers in the double burden of malnutrition, indicating that higher economic standing alone does not necessarily translate into better nutrition outcomes without concurrent improvements in dietary quality and health behaviors.

We have documented that socioeconomic status had a significant relationship with DBM among mother-child pairs in urban Bangladesh. Our research revealed that DBM was more prevalent in households where mothers had a higher education and belonged to the richest SES group. Additionally, the study indicated that as socioeconomic status improved from lower to upper levels, undernutrition decreased while overnutrition increased. This trend reflects the ongoing nutritional transition in urban Bangladesh, where economic growth and lifestyle changes have led to shifts in dietary patterns [50]. Higher-income households often have greater access to processed and energy-dense foods, contributing to rising obesity rates, while lower-income households continue to struggle with undernutrition due to limited access to diverse and nutritious foods. The interaction analysis further revealed that mothers with at least primary education living in upper SES households were more likely to have DBM. This finding highlights the synergistic effect of education and wealth on nutritional outcomes.

Overall, our findings highlight that DBM in urban Bangladesh is not simply a coexistence of two forms of malnutrition, but a reflection of deeper structural and behavioral shifts occurring across households. Recognizing this complexity is essential for designing policy responses that account for both the protective and risk-amplifying roles of socioeconomic advancement, ensuring that interventions remain relevant to Bangladesh’s evolving urban landscape.

### 4.1. Policy Implications

The rapid policy review found that specific actions are in place in the policy documents in Bangladesh that can tackle both undernutrition and all forms of overnutrition. The existing ones are as follows: exclusive breastfeeding to infants for the first 6 months; nutrition counselling during antenatal and postnatal counseling; school feeding programmes, and market regulation for processed foods. While these are in place, proper governance is required to make sure these interventions are in action to address population level DBM.

Even when the mentioned interventions have the potential to address both mother overnutrition and child undernutrition, the policy documents mostly emphasized capacity to improve nutrition status in favor of children, while these interventions can also improve the mother’s nutritional status. For an example: exclusive breastfeeding provides infants with essential nutrition, but it also helps to regulate the mother’s weight gain during the post-partum period. More awareness raising is required for the existing interventions that have the potential to act as double duty actions in terms of the benefits for both the children and mothers, in order to address household level DBM.

Building on our findings, targeted school-based interventions could help mitigate child overnutrition, including nutrition education, promotion of physical activity, and healthier school meal options. Modifications to the National Nutrition Services (NNS) packages such as integrating guidance for maternal nutrition alongside child-focused programmes could serve as double-duty actions, addressing both undernutrition in children and overnutrition in mothers. Strengthening monitoring and enforcement mechanisms for existing programmes, such as market regulations on processed foods and nutrition counseling during ANC/PNC visits, is also recommended to improve effectiveness.

### 4.2. Strengths and Limitations

There are some limitations which need to considered when interpreting the findings. First, the cross-sectional design prevents us from establishing causality between socio-economic factors and DBM. Second, while BMI is widely used for assessing nutritional status, it does not capture body composition differences, which may affect classifications of undernutrition and overnutrition. Third, the national survey was conducted in 2012–2013 in urban settings only, and the findings may not represent the current national burden of DBM in Bangladesh, or capture any changes that occurred over time, particularly following the global pandemic in 2020–2021 [51]. Fourth, the sampling design did not incorporate sub-ward stratification (e.g., slum vs. non-slum neighborhoods) due to the structure of the original national survey. As a result, some intra-urban socioeconomic variation may not be fully represented, although the use of sampling weights likely reduced potential bias from unequal probabilities of selection.

Post-pandemic findings suggest significant shifts in children and adolescent nutrition in Bangladesh, largely due to increased household food insecurity, reduced family income, and extended stay-at-home orders [52,53,54,55]. However, systematic post-COVID national data on school-aged children and adolescents are still scarce. Future surveys should integrate longitudinal measures of nutritional status, including dietary habits, to better explain how DBM evolves in response to socioeconomic shocks and transitions.

To contextualize our findings, recent studies continue to highlight the persistence of DBM in Bangladesh. For instance, Tariqujjaman et al. (2022) reported determinants of DBM among school children and adolescents in urban Dhaka, and Das et al. (2019) described household-level DBM patterns nationally [19,37]. These studies indicate that the issues identified in our data remain relevant today. However, there has not been any baseline data for Bangladesh before the COVID-19 pandemic, and thus our research represents a strong baseline for the burden of DBM in Bangladesh in urban populations, which would allow us to monitor changes following the COVID-19 pandemic. Policy translation would be improved by future analyses in more recent national evidence, including data from the latest Bangladesh Demography and Health Survey and the National Micronutrient Survey.

Despite these limitations, our study has several strengths. Our research findings were derived from population-based samples representative of the study populations at the divisional level, which allows a comprehensive comparative analysis of the regional patterns of DBM in Bangladesh, which is rare to achieve in a low resource setting. Finally, the use of standardized anthropometric measurements and internationally recognized BMI classifications enhanced the reliability of our findings. In future studies, sampling should be expanded to include the newly established city corporation areas as well as semi-urban regions to enhance geographical diversity and generalizability.

## 5. Conclusions

This study provides the first national-level evidence of the double burden of malnutrition among children, adolescents, and mother-child dyads in urban Bangladesh. The findings highlight that DBM is highly prevalent, particularly among younger children and in households with higher maternal education and socioeconomic status, reflecting the ongoing nutritional transition in urban areas. Maternal education emerges as a key determinant of DBM, with higher education improving undernutrition outcomes, which is also associated with increased risk of overnutrition. Regional differences in DBM further highlight the need for targeted interventions tailored to local nutrition contexts. Addressing DBM requires coordinated, double-duty strategies. These include targeted nutrition education for higher-SES families, sustained support for vulnerable households, and integration of BMI screening and counseling into routine child health services. Longitudinal monitoring at both individual and community levels is essential to track emerging trends and inform adaptive responses. Although few policy documents explicitly mention “DBM,” several include implicit “double-duty” actions that could simultaneously prevent undernutrition and curb overnutrition. This suggests that there are opportunities to better align existing nutrition policies with the DBM agenda and incorporate region-specific programming to design scalable and sustainable responses. By capturing these nuanced patterns, this study advances our understanding of DBM in South Asia and provides evidence to inform actionable, context-specific policies.

## Figures and Tables

**Table 1:** Recruitment procedure details.

City Corporations (CC)	Year of Establishment *	Total Number of Wards	Number of Wards Selected	Children Recruited Per Ward	Total Recruited
Dhaka	1864	92	30	20	600
Chattogram	1990	41	30	20	600
Rajshahi	1976	30	30	20	600
Sylhet	2001	27	27	20	540
Khulna	1984	31	30	20	600
Barisal	2002	30	30	20	600
Rangpur	2012	33	30	20	600

* Source: Local Government Division; Government of the People’s Republic of Bangladesh; https://lgd.gov.bd/site/page/a50d48cd-7441-487d-b293-a1d221ba26f8/-, accessed on 10 December 2025).

**Table 2:** Socio-demographic characteristics and socioeconomic status of children and parents (N = 4140).

Characteristics	N = 4140	Percentage (%)
Child information		
Age, Mean (SD)	11 (4)	
Children 5–9 y, *n(%)*	1373	33.2
Adolescent 10–18 y, *n(%)*	2767	66.8
Girls, *n(%)*	2084	50.3
Education, *n(%)*		
Never went to school	275	6.6
Below primary (1–4 grade/pre-school education)	1944	47.0
Completed primary (5–10 grade)	1444	34.9
Completed secondary (11–12 grade)	477	11.5
Parental information		
Mother educations, *n(%)*		
Never went to school	551	13.3
Below primary (1–4 grade)	429	10.4
Completed primary (5–9 grade)	1642	39.7
Completed secondary (10–11 grade)	797	19.3
Completed higher secondary (12th grade or above)	356	8.6
Completed college (Bachelors/Masters)	365	8.8
Father education, *n(%)*		
Never went to school	583	14.1
Below primary (1–4 grade)	394	9.5
Completed primary (5–9 grade)	1334	32.2
Completed secondary (10–11 grade)	684	16.5
Completed higher secondary (12th grade)	399	9.6
Completed college education (Bachelors/Masters)	746	18.0
Father occupations, *n(%)*		
Service	1232	33.4
Manual labor *	794	21.5
Business	1600	43.4
Not engaged in earning ^$^	63	1.7
Mother occupation, *n(%)*		
Housewife	3692	91.0
Service	262	6.5
Manual labor *	52	1.3
Business	51	1.3
Socioeconomic status (SES), *n(%)*		
Lower	721	17.4
Lower-middle	670	16.2
Middle	1071	25.9
Upper middle	721	17.4
Upper	956	23.1

* Manual labor: rickshaw/van puller/transport worker/migrant worker/agriculture; ^$^ Not engaged in earning: Student/unemployed.

**Table 3:** Prevalence of double burden of malnutrition among children and adolescents at population level and variations across socio-demographic characteristics (n = 4140).

Characteristics	n Out of Total (N = 4140)	DBM at Population Level (Children & Adolescents)	
		Prevalence ^1^, %	Unadjusted OR (90% CI)
Overall, %	4140	45.2	
Sex, *n(%)*			
Boys	2056	44.5	1.04 (0.93–1.15)
Girls	2084	43.5	1.0
Age (in years)			
Children (5–9 y)	1373	51.3	1.56 (1.40–1.74) ^$^
Adolescent (10–14 y)	2767	40.3	1.0
Education of children, *n(%)*			
Never went to school	275	45.8	1.26 (0.93–1.70)
Below primary/Pre-school education	1944	44.5	1.20 (0.97–1.43)
Completed primary	1444	44.2	1.18 (0.96–1.46)
Completed secondary	477	40.0	1.0
Mothers’ education, *n(%)*			
Low education (never went to school/below primary)	980	48.3	1.22 (1.04–1.44) ^$^
Completed primary education	1642	42.1	0.96 (0.83–1.10)
Completed secondary and above education	1518	43.1	1.0
Fathers’ education, *n(%)*			
Low education (never went to school/below primary)	977	47.7	1.26 (1.08–1.48) ^$^
Completed primary	1334	44.2	1.10 (0.95–1.26)
Completed secondary	1829	41.8	1.0
Fathers’ occupation, *(n%)*			
Service	1232	44.5	1.01 (0.84–1.21)
Business	1600	42.8	0.94 (0.74–1.11)
Manual labor	794	44.2	1.0
Mothers’ occupation, *(n%)*			
Housewife	3692	43.9	1.0 (0.8–1.24)
Employed **	365	43.8	1.0
Socio-economic status (SES), *n(%)*			
Lower	834	44.4	1.0
Lower middle	974	45.1	1.0 (0.85–1.18)
Middle	759	44.4	1.03 (0.88–1.21)
Upper middle	813	41.2	0.88 (0.74–1.04)
Upper	760	44.6	1.01 (0.85–1.19)
Division, *n(%)*			
Dhaka	282	47.0	1.33 (1.06–1.67) ^$^
Sylhet	257	47.6	1.36 (1.08–1.72) ^$^
Chatttogram	252	42.0	1.09 (0.86–1.37)
Barisal	265	44.2	1.19 (0.94–1.49)
Khulna	240	40.0	1.00 (0.79–1.26)
Rajshahi	284	47.3	1.35 (1.07–1.69) ^$^
Rangpur	240	40.0	1.0

** Employed: Service/business/manual labor; ^1^ Both underweight and overweight/obesity out of total children; ^$^ Level of significance at *p* < 0.1.

**Table 4:** Factors contributing to the double burden of malnutrition among children and adolescents (5–19 years) at population level across divisions (N = 4140).

	Overall	Dhaka	Sylhet	Chottogram	Barisal	Khulna	Rajshahi	Rangpur
	OR (95% CI)	OR (95% CI)	OR (95% CI)	OR (95% CI)	OR (95% CI)	OR (95% CI)	OR (95% CI)	OR (95% CI)
Child age								
5–9 y (Ref: 10–19 y)	1.56 (1.37–1.78)	1.3 (0.93–1.81)	0.88 (0.6–1.29)	1.36 (0.96–1.92)	1.83 (1.29–2.6) ^$^	1.57 (1.09–2.27) ^$^	2.28 (1.6–3.25) ^$^	1.77 (1.22–2.56) ^$^
Wealth index								
Lower	0.97 (0.76–1.24)	0.55 (0.19–1.55)	3.21 (1.16–8.9) ^$^	1.2 (0.56–2.58)	0.64 (0.32–1.29)	1.81 (0.87–3.77)	1.42 (0.67–3.01)	0.5 (0.19–1.31)
Lower middle	1.01 (0.81–1.26)	0.78 (0.42–1.44)	2.06 (1.12–3.8) ^$^	1.31 (0.74–2.31)	0.59 (0.31–1.11)	1.68 (0.87–3.26)	1.54 (0.82–2.89)	0.48 (0.18–1.26)
Middle	1.01 (0.81–1.25)	1.04 (0.61–1.78)	1.74 (1.07–2.82) ^$^	1.29 (0.74–2.24)	0.79 (0.43–1.47)	1.43 (0.75–2.73)	1.1 (0.57–2.09)	0.36 (0.13–1.03)
Upper middle	0.87 (0.71–1.07)	0.59 (0.37–0.93) ^$^	1.06 (0.66–1.71)	0.71 (0.43–1.17)	0.99 (0.56–1.75)	1.39 (0.74–2.6)	1.5 (0.77–2.92)	0.48 (0.16–1.41)
Richest (Reference value)	1.0	1.0	1.0	1.0	1.0	1.0	1.0	1.0
Father‘s education								
Low/below primary *	1.01 (0.84–1.22)	0.7 (0.37–1.34)	0.91 (0.49–1.69)	0.95 (0.53–1.71)	1.57 (0.8–3.05)	0.48 (0.26–0.86) ^$^	0.88 (0.5–1.56)	1.96 (1.11–3.48) ^$^
Completed primary e	1.02 (0.82–1.27)	0.76 (0.45–1.27)	0.92 (0.57–1.48)	1.36 (0.86–2.17)	1.38 (0.8–2.39)	0.59 (0.36–0.97) ^$^	0.88 (0.55–1.4)	1.53 (0.91–2.57)
Completed secondary (Reference value)	1.0	1.0	1.0	1.0	1.0	1.0	1.0	1.0
Mother‘s education								
Low/below primary *	1.05 (0.83–1.32)	1.24 (0.64–2.42)	0.82 (0.45–1.52)	0.96 (0.52–1.77)	0.96 (0.48–1.89)	1.27 (0.68–2.37)	1.35 (0.73–2.47)	0.82 (0.43–1.59)
Completed primary	0.94 (0.78–1.12)	1.26 (0.78–2.06)	0.6 (0.38–0.97)	0.82 (0.51–1.31)	0.95 (0.57–1.57)	1.06 (0.65–1.74)	0.98 (0.6–1.59)	1.1 (0.63–1.94)
Completed secondary (Reference value)	1.0	1.0	1.0	1.0	1.0	1.0	1.0	1.0

* Low education: (never went to school/below primary); ^$^ Statistical significance at *p* < 0.05.

**Table 5:** Prevalence of double burden of malnutrition among children and adolescents at household level (Mother-Child Pair) and variations across socio-demographic characteristics (n = 4140).

Characteristics	n Out of Total (N = 4140)	DBM at Household Level (Mother-Child Pair)	
		Prevalence ^2^, %	Unadjusted OR (90% CI)
Overall, *%*	4140	16.6	
Sex, *n(%)*			
Boys	2056	16.0	1.06 (0.92–1.25)
Girls	2084	15.2	1.0
Age (in years), *n(%)*			
Children (5–9 y)	1373	20.0	1.61 (1.39–1.87) ^$^
Adolescent (10–14 y)	2767	13.4	1.0
Education of children, *n(%)*			
Never went to school	275	19.3	1.22 (0.83–1.79)
Below primary/Pre-school education	1944	26.7	1.86 (1.43–2.42) ^$^
Completed primary	1444	21.8	1.42 (1.08–1.87)
Completed secondary	477	16.4	1.0
Mothers’ education, *n(%)*			
Low education (never went to school/below primary)	980	28.8	1.74 (1.44–2.11) ^$^
Completed primary	1642	24.2	1.38 (1.16–1.64)
Completed secondary and above	1518	18.8	1.0
Fathers’ education, *n(%)*			
Low education (never went to school/below primary)	977	26.9	1.52 (1.27–1.83) ^$^
Completed primary	1334	26.0	1.46 (1.23–1.72 ^$^
Completed secondary and above	1829	19.4	1.0
Fathers’ occupation, *(n = 3626;%)*			
Service	1232	26.3	1.87 (1.49–2.35)^$^
Business	1600	25.1	1.76 (1.41–2.19) ^$^
Manual labor	794	16.0	1.0
Mothers’ occupation *(n = 4057;%)*			
Housewife	3692	23.4	1.01 (0.74–1.30)
Employed **	365	23.6	1.0
Socio-economic status (SES), *n(%)*			
Lower	834	15.6	1.36 (1.07–1.73) ^$^
Lower middle	974	18.0	1.61 (1.28–2.03) ^$^
Middle	759	17.9	1.6 (1.26–2.04) ^$^
Upper middle	813	13.8	1.17 (0.92–1.51)
Upper	760	12.0	1.0
Division, *n(%)*			
Dhaka	282	19.0	1.44 (1.11–1.87) ^$^
Sylhet	257	13.5	0.96 (0.72–1.27)
Chattogram	252	14.3	1.03 (0.78–1.35)
Barisal	265	15.7	1.14 (0.87–1.49)
Khulna	240	16.0	1.17 (0.9–1.53)
Rajshahi	284	16.2	1.18 (0.91–1.55)
Rangpur	240	14.0	1.0

** Employed: Service/business/manual labor; ^2^ OW mothers with UW children compared to counterparts; ^$^ Level of significance at *p* < 0.1.

**Table 6:** Factors contributing to double burden of malnutrition at household level among mother-child pairs across divisions (N = 4140).

	Overall	Dhaka	Sylhet	Chottogram	Barisal	Khulna	Rajshahi	Rangpur
	OR (95% CI)	OR (95% CI)	OR (95% CI)	OR (95% CI)	OR (95% CI)	OR (95% CI)	OR (95% CI)	OR (95% CI)
Child age								
5–9 y (Ref: 10–19 y)	1.64 (1.38–1.95) ^$^	1.83 (1.17–2.86) ^$^	0.95 (0.57–1.6)	1.46 (0.95–2.24)	1.99 (1.22–3.23) ^$^	1.57 (0.97–2.57)	1.96 (1.25–3.06) ^$^	1.24 (0.72–2.14)
Socio-economic status (SES)								
Lower	1.17 (0.83–1.66)	--	2.48 (0.69–8.91)	2.75 (1.05–7.2) ^$^	0.86 (0.29–2.52)	1.36 (0.49–3.83)	1.45 (0.52–4.03)	0.9 (0.25–3.24)
Lower middle	1.41 (1.03–1.93) ^$^	1.43 (0.64–3.21)	2.64 (1.13–6.18) ^$^	2.51 (1.16–5.47) ^$^	0.9 (0.33–2.5)	1.21 (0.46–3.15)	1.45 (0.6–3.51)	0.77 (0.22–2.73)
Middle	1.49 (1.1–2.03) ^$^	1.82 (0.92–3.59)	2.62 (1.32–5.19) ^$^	1.62 (0.73–3.58)	1.6 (0.6–4.22)	1.48 (0.58–3.76)	1.51 (0.61–3.72)	0.08 (0.01–0.8)
Upper middle	1.12 (0.83–1.51)	0.89 (0.47–1.68)	1.12 (0.52–2.38)	1.58 (0.77–3.22)	1.37 (0.53–3.53)	0.75 (0.28–2.03)	1.56 (0.62–3.91)	0.49 (0.11–2.21)
Upper	1.0	1.0	1.0	1.0	1.0	1.0	1.0	1.0
Father‘s education								
Low/below primary *	1.14 (0.9–1.46)	1.14 (0.48–2.68)	1.38 (0.61–3.16)	1.03 (0.5–2.14)	1.46 (0.58–3.68)	0.38 (0.17–0.82) ^$^	0.68 (0.33–1.41)	0.89 (0.38–2.07)
Completed primary education	1.11 (0.82–1.49)	1.06 (0.54–2.07)	1.03 (0.53–2.03)	1.47 (0.82–2.63)	1.69 (0.8–3.57)	0.56 (0.29–1.06)	0.73 (0.41–1.31)	1.02 (0.49–2.14)
Completed secondary education	1.0	1.0	1.0	1.0	1.0	1.0	1.0	1.0
Mother‘s education								
Low/below primary *	1.27 (0.93–1.74)	0.53 (0.2–1.4)	0.73 (0.32–1.66)	1.15 (0.54–2.44)	1.59 (0.6–4.18)	4.26 (1.81–10) ^$^	1.67 (0.75–3.72)	0.41 (0.15–1.13)
Completed primary education	1.2 (0.93–1.53)	1.22 (0.64–2.31)	0.53 (0.27–1.05)	0.9 (0.49–1.64)	1.71 (0.82–3.55)	1.72 (0.84–3.54)	1.5 (0.79–2.83)	1.03 (0.46–2.31)
Completed secondary education		1.0	1.0	1.0	1.0	1.0	1.0	1.0

* Low education: (never went to school/below primary); ^$^ Statistical significance at *p* < 0.05.

**Table 7:** Overview of Bangladeshi nutrition policies, malnutrition focus, double-duty interventions, and gaps.

Policy/Plan	Forms of Malnutrition Addressed	Potential Double-Duty Interventions	Operational Gaps/Limitations
National Health Policy, 2011	Undernutrition (women, pregnant mothers, children)	None explicitly; focus on undernutrition	Overweight, obesity, DBM, double-duty actions not mentioned; limited relevance for emerging overnutrition
National Nutrition Policy, 2015	Undernutrition, emerging overweight/obesity, nutrition-related NCDs	Exclusive breastfeeding, complementary feeding, iron/folic acid supplementation, nutrition education, food marketing regulation	DBM and double-duty actions not explicitly recognized; interventions mostly single-form; weak integration for dual outcomes
Second National Plan of Action for Nutrition (2016–2025)	Undernutrition, overweight/obesity, nutrition-related NCDs	Infant and young child feeding (IYCF), antenatal/postnatal care counseling	No explicit DBM or double-duty framework; limited household-level coverage; monitoring focused on undernutrition
National Plan of Action for Adolescent Health Strategy (2017–2030)	Undernutrition, overweight/obesity	School feeding, nutrition and health education, screening and counseling for under- and overweight adolescents	DBM not explicitly framed; requires strong school-health coordination; risk of fragmented implementation
Bangladesh National Strategy for Maternal Health (2019–2030)	Undernutrition (low BMI, stunting, food insecurity)	ANC/PNC nutrition counseling, iron/folic acid supplementation	Overweight/obesity not addressed; DBM not recognized; household-level dual burden interventions limited
National School Meal Policy 2019	Undernutrition, overweight (children)	School meals providing calories and micronutrients	DBM and double-duty actions not explicitly addressed; nutrition balance may not prevent obesity; limited evaluation mechanisms
Bangladesh National Food and Nutrition Security Policy: Plan of Action (2021–2030)	Undernutrition, overweight/obesity, nutrition-related NCDs	Nutrition education in schools, food labeling/regulation, promoting fruit/vegetable consumption	DBM not explicitly framed; multi-sectoral coordination needed; monitoring for dual outcomes may be weak

## Data Availability

The original contributions presented in this study are included in the article/Supplementary Materials. Further inquiries can be directed to the corresponding author.

## Supplementary Materials

The following supporting information can be downloaded at: https://www.mdpi.com/article/10.3390/nu18010135/s1, Figure S1: Marginal effect of socio-demographic characteristics on double burden malnutrition at population level; Figure S2: Marginal effect of socio-demographic characteristics on double burden malnutrition at household level; Table S1: Population (aged 5–19 years), sample size, and sampling weights for each City Corporation.

## Abbreviations

The following abbreviations are used in this manuscript:

NNS	National Nutrition Service
DBM	Double Burden of Malnutrition
BMI	Body Mass Index
WHO	World Health Organization
IYCF	Infant and young child feeding
ANC	Antenatal Care
PNC	Postnatal Care
NCD	Non-communicable diseases
