# Supplementary material for: Sociodemographic Factors Attributed to the Double Burden of Malnutrition in Urban Bangladesh
Source: Nutrients. 2025 Dec 31;18(1):135. doi: 10.3390/nu18010135 (PMC12787961; doi:10.3390/nu18010135)
Supplement: Supplementary file 1 [file nutrients-18-00135-s001.zip › nutrients-3992777-supplementary.pdf]

Supplementary analysis S1

Weighting procedure: Post-stratification weights

Step 1: Construction of base weights

Because the study used equal sample allocation across the seven City Corporations despite large differences in the underlying population of children and adolescents (aged 5–19 years), we constructed post-stratification sampling weights. For each City Corporation (CC), the base (raw) weight was calculated as:

$$Weight_{CC} = \frac{\text{Estimated population aged 5} - \text{19 in CC}}{\text{Total recruited from that CC}}$$

Step 2: Normalization of weights

To stabilize variances and maintain comparability across models, raw weights were normalized to have a mean of 1, using the following standard normalization formula:

$$Normalized\ weight_i = \frac{Raw\ weight_i}{Mean\ of\ all\ raw\ weight}$$ (i=1,…,7 City Corporations)

The resulting normalized weights were used in all analyses

Step 3: Application of Post-Stratified Weights

Post-stratification weights were applied to align the sample distribution with the actual population distribution of children and adolescents across the seven City Corporations. This approach corrects the disproportionate allocation caused by sampling equal numbers from cities with widely varying population sizes. All weighted analyses additionally accounted for the complex survey design, including stratification by City Corporation and clustering at the ward level.

Table S1. Population (aged 5–19 years), sample size, and sampling weights for each City Corporation

City Corporation	Population 5–19 yrs (2011)	Sample recruited	Raw weight	Normalized weight
Dhaka	2,155,410	600	3592.35	3.955
Chattogram	890,733	600	1484.56	1.635
Rajshahi	139,969	600	233.28	0.257
Sylhet	173,431	540	321.17	0.354
Khulna	220,086	600	366.81	0.404
Barisal	116,183	600	193.64	0.213
Rangpur	102,803	600	171.34	0.189

Supplementary analysis-S2

Figure S1: Marginal effect of socio-demographic characteristics on double burden malnutrition at population level

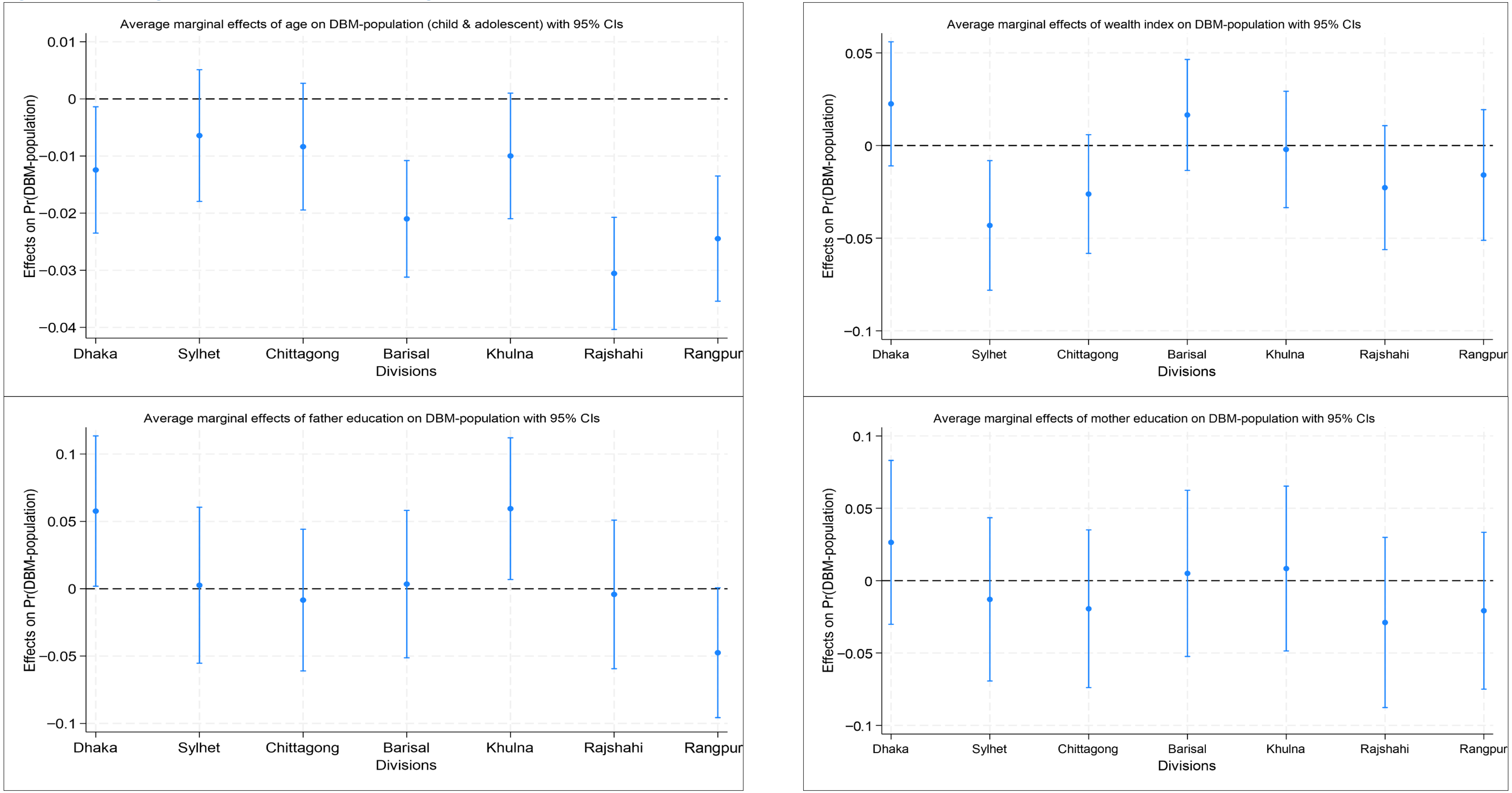

Average marginal effect of socio-demographic characteristics on DBM at population level across divisions. Vertical lines indicate seven divisions and horizontal line indicates marginal effect of characteristics; DBM=double burden of malnutrition.

Figure S2: Marginal effect of socio-demographic characteristics on double burden malnutrition at household level

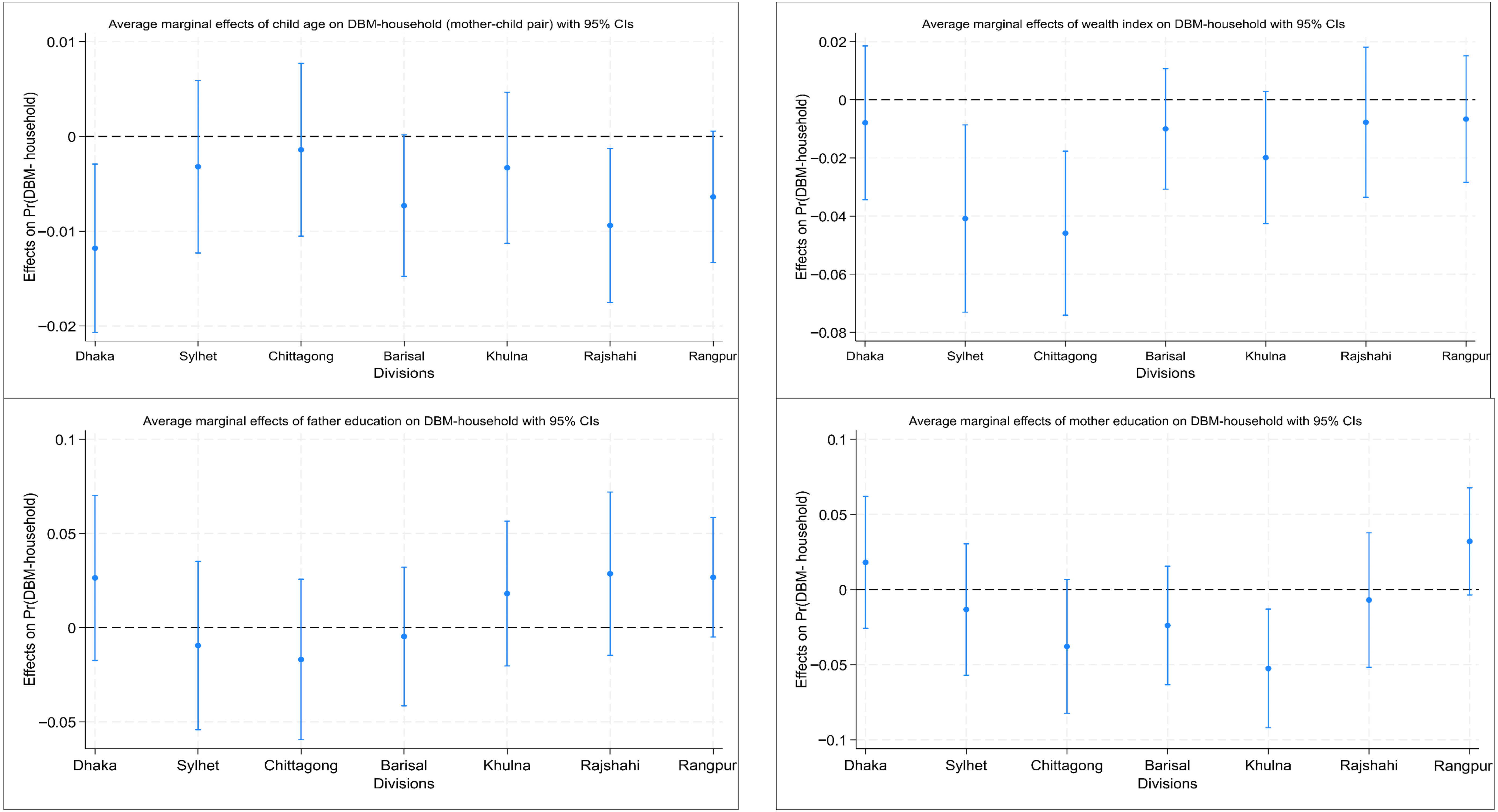

Average marginal effect of socio-demographic characteristics on DBM at population level across divisions. Vertical lines indicate seven divisions and horizontal line indicates marginal effect of characteristics; DBM=double burden of malnutrition.
